# Molecular cytogenetic analysis and clinical manifestations of a case with *de novo *mosaic ring chromosome 7

**DOI:** 10.1186/1755-8166-4-5

**Published:** 2011-02-08

**Authors:** Li-Ping Tsai, Kuei-Fang Lee, Jye-Siung Fang, Ingrid Y Liu

**Affiliations:** 1 Department of Pediatrics, Buddhist Tzu Chi General Hospital, Taipei Branch, 289, Jianguo Rd., Sindian City, Taipei County 231, Taiwan; 2 Department of Medicine, Tzu Chi University, 701, Sec 3, Chunyang Rd., Hualien 970, Taiwan; 3 Laboratory for Cytogenetics, Centre for Genetic Counselling, Buddhist Tzu Chi Hospital, 701, Sec 3, Chunyang Rd., Hualien 970, Taiwan; 4 Department of Molecular Biology and Human Genetics, Tzu Chi University, 701, Sec 3, Chunyang Rd., Hualien 970, Taiwan

## Abstract

**Aim:**

Clinical and molecular cytogenetic investigations of a newborn girl exhibiting facial dysmorphism with developmental delay.

**Methods:**

Phenotypic evaluation was first applied to examine the proband's developmental status. Computed tomography and colour transcranial Doppler were used then to investigate her brain structure and function. Subsequently, chromosomal abnormalities were examined by karyotyping and fluorescent *in situ *hybridization was performed to investigate size of fragments lost at the two distal ends of the ring chromosome 7. In addition, multicolour banding was applied to rule out structural rearrangement occurs in between the ring chromosome 7.

**Results:**

The proband was born with mosaic supernumerary ring chromosome 7, without a normal karyotype detected in the peripheral blood lymphocytes. The distal arm of chromosome 7p (at least 255 kb from the telomere) was part of an extra ring chromosome 7. In addition, the distal arm of 7q, at least 8 kb from the telomere, was missing. There was no other chromosomal rearrangement detected by multicolour banding.

**Interpretation:**

This is the 19^th ^reported case of complete ring chromosome 7 mosaicism and the first survived case with mosaic supernumerary ring 7 without a normal karyotype detected in the peripheral lymphocytes.

## Introduction

Ring chromosome 7 is a rare chromosome anomaly that leads to variable phenotypes. The first two cases were described by Zackai *et al *in 1973 [1]. A total of 18 cases with complete ring chromosome 7 have been reported to date worldwide [1-17]. Ring chromosomes are often unstable during mitosis; therefore, it is common to find a ring chromosome in only a portion of all cells analyzed (mosaicism). The other cells in an individual, with a ring chromosome, are usually monosomic or demonstrate partial-trisomy with a small ring [18]. Here we present the 19^th ^case of complete ring chromosome 7 mosaicism. This is the first survived case with complete supernumerary ring 7, without a normal karyotype detected in the peripheral lymphocytes. The phenotypic expression of patients with ring chromosome 7 is variable; most patients demonstrate developmental delay, mental retardation, microcephaly, and dermatological abnormalities including cafe-au-lait spots, nevus flammeus and dark pigmented nevi (for review, please see [7]). The variable phenotypes may result from the variable size of the deleted chromosomes at the terminal segments, ring instability, and/or the level of mosaicism. The case presented here had microcephaly, hypotelorism, choanal stenosis, and speech delay, without any dermatological abnormalities detected to date.

## Methods

### Patient and Clinical examination

**Figure 1:**
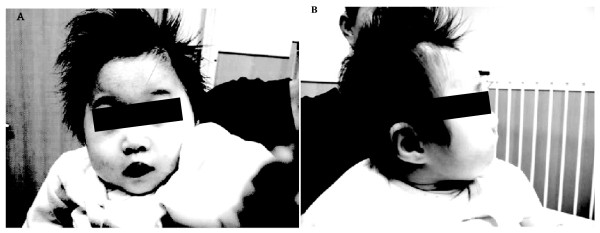
**Pictures taken from the front (A) and from the side (B) of the proband at her age of one year and 10 months old**. Facial dysmorphism including hypotelorism, midface hypoplasia and high arch palate were noted. Choanal stenosis was suspected because of noise nasal breathing since birth.

A female child with microcephaly (Figure 1 A and 1B, pictures taken at one year and 10 months of age) and respiratory distress was referred to the genetic counselling at the department of Pediatrics at Tzu Chi General Hospital in Taipei County, Taiwan. The proband was the first child of non-consanguineous parents. The Vietnamese mother was 23 at delivery, and the Taiwanese father was in his 40 s. The proband was born by caesarean section with a birth weight of 2410 gm, length 42 cm and head circumference of 30 cm, all fall below the third percentile for gestational age. Facial dysmorphism was impressed, including hypotelorism, midface hypoplasia and high arch palate. Choanal stenosis was suspected because of noise nasal breathing since birth. Physical examination also detected subcutaneous syndactyly of 3^rd ^and 4^th ^toes. Computed tomography (CT) and transcranial Doppler were directed due to microcephaly. CT of the brain showed mild stepage, widening of the upper part of the right lambdoid suture and mild scalp thickening along the course of the right lambdoid suture. The pediatric brain echo revealed normal ventricles and choroid plexus pulsation; however, a hypoechoic cyst-like lesion was noted next to the quadrigeminal plate. Speech delay and psychomotor retardation was noted during clinical evaluation at thirteen months old. The body weight of 5.4 Kg, height of 61 cm and head circumference of 35.2 cm still fell far below the third percentile on growth curve.

### Metaphases preparation and florescent in situ hybridization (FISH)

Metaphases slides were prepared from lymphocyte cultures and G-banded for karyotyping using a standard protocol [19]. Five metaphase slides then were incubated in 2 × SSC (pH7.0) prewarmed to 37°C for 30 min for FISH analysis. After that, slides were dehydrated sequentially in a 70%, 85% and 100% ethanol series for 2 min each and then air dried. The metaphase slides were next denatured for 5 min in denaturant solution (70% formamide/2 × SSC) in a 73°C water bath inside of a coplin jar, and then dehydrated in serial ethanol for 2 min each. Subsequently, 10 μl of denatured probes (G31340 for chromosome 7q and G31341 for 7p, Cytocell, Ltd, Cambridge, UK) were added to the metaphase slides and then covered with a cover glass; the slides were placed in a prewarmed humidified box and incubated overnight at 42°C for hybridization. The next day, the cover glasses were removed immediately and the slides were washed with 0.4 × SSC/0.3% NP40 three times for 2 min each, and then air dried in the dark. Hybridization areas were counterstained with 20 μl DAPI (Vysis Inc, IL, USA) and then examined under a fluorescence microscope.

### Multicolor banding

Two metaphase slides were prepared for multicolour banding FISH analysis, Multicolor banding FISH probes for chromosome 7 (XCyte 7 24 μl D-0207-024-MC, XCyte 7 24 μl D-0207-024-MC) were hybridized to metaphases using standard FISH protocol described above and viewed under the fluorescent microscope (Axioskop 40, Carl Zeiss Inc., Germany).

## Results

**Figure 2:**
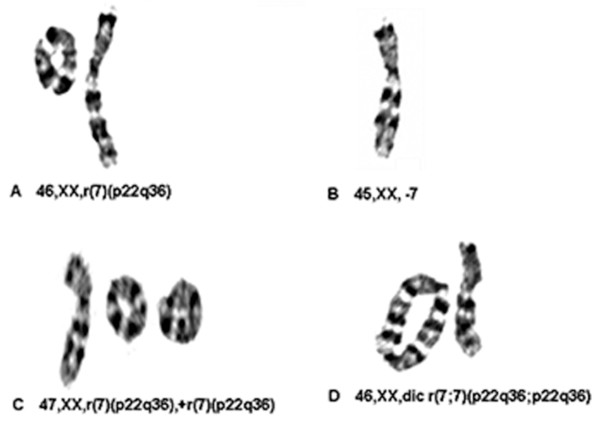
**Mosaic karyotypes of the proband: Out of 100 analyzed cells, 46, XX,r(7) (p22q36) (A) was detected in 75 cells (75%); 45,XX, -7 (B) in 12 cells (12%); 47,XX,r(7)(p22q36),+ r(7)(p22q36) (C) in five cells (5%); and 46, XX, dic r (7;7)(p22q36;p22q36)(D) in four cells (4%)**.

**Figure 3:**
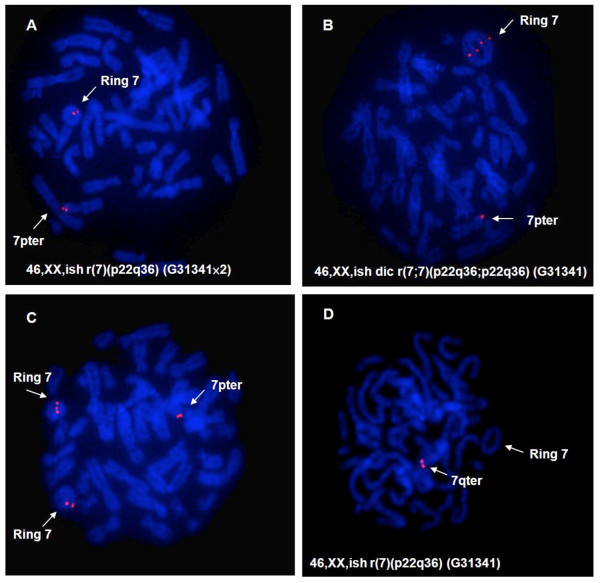
**Flourescent In Situ Hybridization (FISH) using 7p and 7q sub-telomere probes: FISH with 7pter probe indicates subtelomeric region at least 255 kb from telomere remained intact in all rings (A, B and C)**. However, the 7qter probe indicates that at least 8 kb from telomere was missing in the ring (D).

Chromosome analysis of the peripheral lymphocytes revealed a mosaic karyotype. Out of 100 cells analyzed, a 46,XX,r(7)(p22q36) (Figure 2A) complement was observed in 75 cells (75%); 45,XX, -7 (Figure 2B) in 12 cells (12%); 47,XX,r(7)(P22q36),+r(7)(p22q36) (Figure 2C) in five cells (5%); 46,XX,dic r(7;7)(p22q36;p22q36)(Figure 2D) in four cells (4%); 47,XX,r(7), +dicr(7;7)(p22q36;p22q36), and 47,XX,r(7),+inv(7)(p11.1-p22::p36-q11.1) in two cells (2%). A total of 13% of the examined cells revealed a supernumerary ring 7. Skin fibroblast analysis was planned but the family did not return for further evaluations. A subtelomere specific probe was hybridized to metaphase cells by fluorescent *in situ *hybridization (FISH) to determine how much of the distal region of chromosome 7 remained intact in the ring chromosome 7. The results showed that the distal segment of chromosome 7p (at least 255 kb from telomere) appeared to be present in all of the ring chromosome 7 fragments (Figure 3A-C). However, the distal segment of chromosome 7q (at least 8 kb from telomere) was missing (Figure 3D).

**Figure 4:**
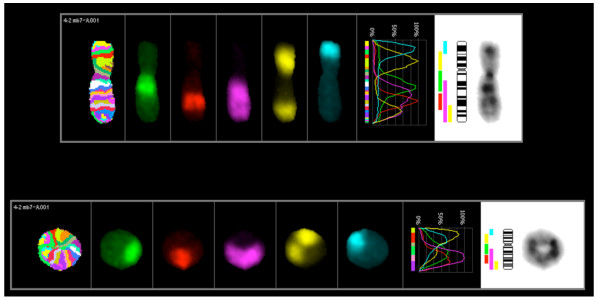
**Multicolor banding of normal and ring chromosome 7: Multicolor banding probes that specifically detect various regions along chromosome 7 were hybridized to metaphases; no rearrangement was observed in normal ring 7 chromosomes**.

Multicolour banding was performed to determine whether a micro-rearrangement occurred in the ring chromosome 7. Multicolor chromosome 7 banding probes that specifically detect various regions along chromosome 7 were hybridized to metaphases and viewed under the fluorescent microscope. No chromosome rearrangement was detected (Figure 4).

## Discussion

A ring chromosome is formed by telomere-to-telomere fusion with minimal or no loss of genomic material. Ring chromosomes are often unstable during mitosis; as a result, it is common to find a ring chromosome in only a portion of cells (mosaicism). The other cells in the individual are usually monosomic or demonstrate partial-trisomy. Some cells lose the ring chromosome and some cells maintain a supernumerary ring chromosome in which the ring is usually small and composed mostly of the centromere and a small amount of euchromatic material [19].

The karyotype of the patient presented in this report was complicated and unique. There was no normal karyotype detected in the peripheral blood lymphocytes. Monosomy 7, one complete ring, supernumerary large rings, and duplicated rings of chromosome 7, were detected. Most previous reported cases of supernumerary ring chromosome 7 showed a majority of cells with a normal karyotype, in addition to some cells with a partial trisomy containing small rings [20-26]. Here we present a newborn girl that survived with a mosaic karyotype with a complete supernumerary ring chromosome 7 and a few monosomy 7 cells, without any cell with normal karyotype detected in peripheral lymphocytes. All large rings have an intact 7p G31341 region with the 7q telomere absent (8 kb from telomere). According to the human chromosome 7 view map (http://www.ncbi.nlm.nih.gov) of the National Centre for Biotechnology Information (NCBI), there are no functional genes located within the missing distal region of 7q. There might be a microdeletion at the 7p telomere; however, no commercial probe available yet to detect a missing segment in this region.

In a previous report of two cases with complete trisomy ring 7 the infants were stillborn [27,28]. In the present case, a majority of cells (75%) were composed of 46, XX, r(7), and all of the large rings appeared to be stable; this is likely associated with the patient's survival. However, it is also possible that other tissues, beside the peripheral lymphocytes, had a normal karyotype; but no other tissue samples were available for analysis. The influence of gene dosage effects resulting from monosomy chromosome 7 (12%) and supernumerary ring chromosome 7 (13%) were likely the cause of the abnormal clinical characteristics in the patient reported here. We also assumed uniparental disomy (UPD) of chromosome 7 was associated with the proband's clinical manifestation, however, further examination for UPD was not possible due to relocation of this family.

The clinical expression of ring chromosome 7 is variable. Nevertheless, most cases share some characteristics such as: mental retardation, growth deficiency, mild microcephaly, facial asymmetry, hypertelorism, abnormal palpebral fissures, small ears, limb and skeletal anomalies, and skin lesions, including nevus flammeus, dark pigmented nevi and café-au-lait spots [7,10]. Our patient had some of these findings e.g. growth retardation, microcephally. However, no pigmented nevi or café-au-lait spots were observed at the time of evaluation. In addition, speech delay was noted. These findings suggest that some of the genes located within the affected region of chromosome 7 are associated with the cognitive abilities needed for speech development and might illustrate a dosage-dependent pattern.

In summary, we present the first case of survival with a mosaic and supernumerary complete ring chromosome 7, without a normal karyotype detected in peripheral blood lymphocytes. The high percentage (75%) of cells with a stable 46,XX, r(7), the absence of a macro-deletion and rearrangement within the ring chromosome 7 are likely the major reasons accounting for the viability of this patient.

## Consent

Written informed consent was obtained from the parents of the patient for publication of this case report and accompanying images.

## Competing interests

The authors declare that they have no competing interests.

## Authors' contributions

L-PT carried out pedigree analysis, clinical examination and diagnosis of this family, drafted "Patient and clinical examination" section. K-FL carried out cytogenetic and molecular cytogenetic experiments. J-SF carried out analysis of cytogenetic studies and interpretation of karyotype. IYL conceived of the study, and participated in its design, coordination and manuscript writing. All authors read and approved the final manuscript.
